# Supplementary material for: Are carbon emissions trading and green financial instruments synergistic? -Comprehensive quantitative research based on content analysis
Source: PLoS One. 2024 Mar 7;19(3):e0298601. doi: 10.1371/journal.pone.0298601 (PMC10919601; doi:10.1371/journal.pone.0298601)
Supplement: S1 Data — (ZIP) [file pone.0298601.s001.zip › Data/地级市Z_F工作报告.zip!/░▓╗╒/═¡┴Ω╩╨/└┤╫╘╣1⁄2╓┌║┼í╛┬φ┐╦╩2╛▌═°í┐_2019.docx]

各位代表：

　　现在，我代表市人民政府，向大会报告政府工作，请予审议，并请各位市政协委员和列席人员提出意见。

**一、奋斗2018，夯基筑路、稳健起航**

　　刚刚过去的一年，是贯彻党的十九大精神开局之年，也是本届政府起步之年。面对复杂严峻的宏观环境和压力叠加的多重挑战，全市人民坚持以习近平新时代中国特色社会主义思想为指导，在省委、省政府和中共铜陵市委的坚强领导下，坚持稳中求进工作总基调，按照高质量发展要求，以供给侧结构性改革为主线，全面落实中央“六稳”部署，深入实施五大发展行动计划，改革创新，攻坚克难，基本完成了市十六届人大一次会议确定的目标任务，现代化幸福美丽新铜陵建设迈出坚实步伐。

　　预计，全年实现生产总值1220亿元，增长5%左右；财政收入180.5亿元，增长7.6%；固定资产投资606亿元，增长8.5%；社会消费品零售总额371亿元，增长8.5%；城镇、农村居民人均可支配收入36212元、14341元，分别增长8.8%、9.1%；节能减排实现年度控制目标。

　　过去的一年，我们立足换届新起点，以奋发有为的姿态、一往无前的状态，日夜兼程思进求变，风雨无阻接续奋斗。这一年，我们在创新发展上先行先试，强化科技引领，推动产业升级，资源型城市转型模式成为全国典型案例；我们在协调发展上群策群力，义无反顾支持枞阳发展，区划调整积极效应逐步显现，“一江两岸”融合联动，一县三区竞相发展的氛围越来越浓；我们在绿色发展上坚定坚决，着力打造水清岸绿产业优铜陵样板，铜陵蓝、铜陵绿成为城市最亮色；我们在开放发展上加力加速，皖中南B型保税物流中心获省政府批准申报，正式加入长三角城市经济协调会和产业创新城市联盟；我们在共享发展上尽心尽力，上榜全国小康城市百强，人民群众获得感幸福感安全感持续增强。

　　一年来，主要做了以下工作：

　　（一）打底层调结构，高质量发展蓄势积能。坚持质量第一、效益优先，保持定力，精准发力，推动经济建设行稳致远。新旧动能加速转换。矢志培育新动能，勇攀产业“中高端”，产业结构持续优化。制定实施“1365”工业转型升级行动方案，电子材料及新型元器件入列省第三批重大新兴产业工程，战略性新兴产业产值占规上工业比重居全省第2位；实施亿元以上技改项目72个，新增省级名牌和工业精品19个，工信领域行业标准领跑全省。现代服务业升级发展，大通古镇获批省级服务业集聚区，铜官区国家服务业综合改革试点稳步实施。数字经济茁壮生长，与科大讯飞建立成长性战略合作伙伴关系，“城市超脑”一期工程启动实施。“工业大脑”成为阿里系国内首个区域性工业大数据平台，在杭州云栖大会上向全球发布，铜官数谷建成并投入运营。新业态新模式不断涌现，“昆仑决”赛秀引爆全城，陆游房车、科讯金服、光大银行抢滩入驻，合作组建长三角产业创新基金。在全省率先成立电商企业联盟，艺福江南等电商企业异军突起，网上零售额增长50%以上。现代农业加快成长，新增各类新型农业经营主体465家，农产品加工业总产值突破230亿元，枞阳县成为中央财政农作物秸秆综合利用试点县。发展潜能加快汇集。强化投资拉动，新开工亿元以上项目133个、竣工108个，完成投资400亿元以上。庐铜铁路建成运营，有色“奥炉”改造、北大青鸟高性能陶瓷电路板等项目陆续投产，引江济淮枞阳段、旭创高速光通信模块等项目开工建设。提振实体经济，深入开展“四送一服”双千工程，创新建立与铜陵有色定期联席会制度，出台促进民营经济发展“28条”，降低企业成本36亿元，蓝盾光电子完成IPO备案辅导，累计直接融资135亿元。规上工业利润增幅居全省第5位，工业经济效益综合指数全省第一。创新创业加油助力。深度融入省“四个一”创新主平台，全社会研发投入占比居全省第4位，每万人发明专利拥有量9.5件。新认定国家高新技术企业67家、“专精特新”企业50户，新增国家级企业技术中心、院士工作站3家。精达集团跻身国家技术创新示范企业行列，耐科科技成为全国制造业单项冠军，中国特检院铜陵技术中心、市标准化研究中心挂牌。以前所未有的力度支持创业就业人员购租房落户，新引进高层次科技人才团队14个，入列省级团队4个。新增市场主体突破1万户，总量迈入“10万+”时代。

　　（二）破瓶颈创优势，改革开放活力迸发。以实际行动庆祝改革开放40周年，逢山开路，遇水架桥，坚定将改革开放进行到底。全力突破重点领域改革。系统组织改革攻坚，完成市委确定的52项改革任务。部分县区行政区划调整顺利实施，郊区更名即将获国务院批准，拥江发展开辟新里程。开发园区优化整合率先完成，市示范园区整体并入铜陵经开区，调整滨江工业园管理体制，试行工业项目“承诺即开工”、“多评合一”，社会事务顺利移交，开发园区减负松绑、轻装前行。铜化集团增资扩股有序实施，普济圩农场完成集团化改造，“三供一业”分离移交基本完成。新一轮社区治理改革获评首届长三角城市治理最佳实践案例奖，居委会特别法人、准物业服务、社区规划师等创新举措全省推广。紧密型医联体改革试点做法得到国务院医改领导小组充分肯定。市县乡三级新税务机构全部挂牌，公共资源、公积金管理实现市县一体化。完成83个村“三变”改革试点，“共享农房”改革初见成效。全面拓展对外开放合作。深化区域合作，加强与北京、上海、深圳等“头部”资源对接，成功举办第四届中国县域电商大会暨安徽省网商大会、第四届铜基新材料国际论坛等大型节会活动，枞阳浮山与台湾阿里山缔结“姊妹山”，铜陵的“朋友圈”越来越大。“双招双引”成效明显，亿元以上省外到位内资575亿元，增长5%；实际利用外商直接投资3.1亿美元，增长15.2%。进博会全省最大单—有色铜精矿进口项目成功签约，外贸进出口总额增长13.9%，总量居全省第3位。全心打造“四最”营商环境。纵深推进“放管服”改革，“一网、一门、一次”改革取得重要突破，“证照分离”营业执照全省首发，“减证便民”市级申请材料精简60%，“最多跑一次”事项占比99.92%，企业设立压缩到1个工作日、不动产登记3个工作日、建设项目施工许可审批40个工作日内完成，市政务服务中心成为全省“最佳服务大厅”。

　　（三）克难点补短板，“三大攻坚战”战果初现。坚持把打好“三大攻坚战”作为必须攻克的难事、必须跨越的关口，大力度实举措推进。防范化解重大风险稳准出招。建立健全风险防范机制，金融风险牢牢守在可控区间。研究制定防范化解债务风险专项方案，累计争取新增债券93亿元，置换政府债务161.6亿元，实现存量政府债务置换全覆盖。开展互联网金融风险专项整治行动，核销处置银行不良贷款12亿元，部分重点企业风险“排雷”取得积极进展。脱贫攻坚尽锐出战。深入开展“重补促”专项整改行动，全力打响“四季攻势”，2.6万贫困人口达到脱贫标准、33个贫困村满足出列条件。深入实施脱贫攻坚“十大工程”和扶贫开发“十项举措”，特色种养业扶贫“万千工程”全省首推，覆盖率90%以上。创新就业扶贫“421模式”，招募就业扶贫基地86家，建成就业扶贫驿站55个。试行非贫困人口大病补充医疗保险，因病致贫防范机制更加健全。污染防治重拳出击。铁腕推进环保督察反馈问题整改，顺利通过中央“回头看”考验。全面打好蓝天、碧水、净土保卫战，实施重点污染防治项目488个，以雷霆之势严厉打击超限超载，集中整治餐饮油烟污染，强力实施冥币纸钱禁烧，PM10、PM2.5平均浓度下降15.1%、17.4%，分别居全省第6、第3位，优良天数比例全省第三，铜陵空气质量改善幅度居全国169个重点城市第13位，“蓝天白云、繁星闪烁”渐成常态。全面落实河（湖）长制，率先实施沿江1公里区域重化工企业综合整治，完成惠溪河、狮矿大沟黑臭水体治理，迁建市三水厂取水口，地表水国控断面水质考核全部达标。深刻汲取“10·12”输入性危废固废偷倒案件教训，开展“清废行动”，135个固废问题全部整治到位。深入推行林长制，创新实施矿山山长制，人工造林1.1万亩，改造提升绿化面积205万平方米，矿山复绿1950亩，青山常绿、碧水长流绘成风景。

　　（四）强统筹提品质，城乡面貌喜展新颜。坚持城乡一体、建管并重，加速建设宜居宜业铜都。智慧城市破题起势。抢占人工智能潮头，加快新型智慧城市建设，全民健康、环境监测、社会治安防控等一批“智慧+”项目积极推进，市级电子政务外网改造升级率先启动，“易铜陵”APP新版上线，社管信息化“六易平台”案例获中国政府信息化管理创新奖，市政府网站绩效考评进入全国50强。功能品位整体提升。交通路网四通八达，“最美连心路”国道347全线通车，省道221大通段、省道217改造工程主体完工，国道236改建工程、省道335路基贯通。新建成“四好农村路”238公里。开通枞阳至市区等公交线路8条，投放清洁能源公交车166辆，交通一卡通、移动支付便捷惠民，共享单车自由穿梭。西湖新区提速建设，十五中西湖校区、师范附小新校区、邻里中心集中开工，一中西湖校区二期建成，西湖湿地升格4A级景区。市政设施不断完善，改建供水管网30公里、雨污水管网25公里，铺设燃气管道25公里，整治微循环道路7条，改造老旧小区10个。大通、横港段岸线整治全面完成，长江岸线颜值更高。乡村环境愈加靓丽。建成美丽乡村省级中心村23个、市级中心村15个、特色自然村8个。一体化推进农村环境“三大革命”，改造卫生厕所2.5万户、农村危房1819户，完成11个乡镇政府驻地污水处理设施建设，解决2.1万农村居民饮水安全问题，农村正在变成可以到达的“诗与远方”。

　　（五）惠民生促和谐，群众福祉持续攀升。把民之所望作为施政方向，发力“七有”领域，让广大群众共享多享改革发展成果。民生实事普惠延伸。高标准实施38项民生工程，财政民生投入占比达82.3%。新增城镇就业2.84万人，登记失业率2.81%。城乡低保、居民养老金不断提标，工伤保险省级统筹全面完成。积极构建多层次住房供给和保障体系，房地产市场总体平稳。改造棚户区1.2万户，宝山区域整治取得重大进展，比学赶超、合力攻坚的征迁局面加速形成。公共资源优质供给。大力实施“四名工程”，新改建公办幼儿园16所，义务教育“全面改薄”顺利完成。探索建立“快乐三点半”课后服务机制，更有温度的铜陵教育得到社会广泛好评。家庭医生签约服务提质扩面，基层卫生服务“两卡制”示范全省。铜都健儿勇夺省运会金牌40枚，创历史新高。创建国家公共文化服务体系示范区高水平通过验收，枞阳方园开园，中国数字铜博物馆、数字文化馆、非遗展示馆建成开放，“月读公社”引领阅读风尚。顺利通过全国文明城市测评、国家卫生城市复审，入选中国好人7人、安徽好人9人，彰显大爱的胡晨当选“全国最美退役军人”。社会治理精细运作。深入开展“深重促”专项行动，退役军人服务管理水平有效提升，教师群体待遇依法依规兑现。扎实推进历史遗留问题化解，多个问题楼盘有效处置。安全生产形势平稳向好。“非洲猪瘟”疫情防控有力，食品药品安全不断巩固。重拳扫黑除恶，群众安全感居全省第4位，平安法治铜陵建设保持全省先进。村（居）民委员会换届选举圆满完成。国防动员建设稳步提质，全省双拥模范城实现“八连冠”。社会科学、新闻出版、广播影视、文学艺术、统计、供销、气象、地震、人防、消防、应急管理等工作取得新成绩，外宣、外事侨务、对台、港澳、民族宗教、妇女儿童、残疾人、红十字等事业实现新进步。

　　过去的一年，我们坚持在善政中求善治，持续加强政府自身建设，全力打造忠诚、高效、卓越、法治、廉洁的“铜都施工队”。深入推进“两学一做”学习教育常态化制度化，扎实开展“讲严立”专题警示教育，推动政府系统全面从严治党向纵深发展。修订政府工作规则，出台首部实体性政府规章，完善重大决策合法性审查机制，组建新一届政府法律顾问团和重大决策专家库。自觉接受人大政协和社会各界监督，办结人大代表建议204件、政协提案336件。严格贯彻中央八项规定精神和省、市委实施细则，“三公”经费支出持续下降。深入推进政府系统党风廉政建设，强化“一岗双责”，狠抓省委巡视反馈问题整改，严肃查处“四风”和腐败问题，营造了风清气正、干事创业的良好政治生态。

　　各位代表！2018年是铜陵历史上不寻常、不平凡的一年，我们先后经受了“环保风暴”多轮洗礼，经历了省委巡视、信访维稳等多重考验，顶住了经济下行、转型阵痛等多种压力，负重奋战，愈战愈勇。一年的艰苦实践，生动诠释着铜陵发展的律动和轨迹，累积的经验弥足珍贵。我们深深体会到，做好政府工作：必须始终坚持发展为上。发展是永恒的主题。任何时候都要把推动发展鲜明地镌刻在心中，用发展的眼光看问题，凭发展的办法解难题，奋力在高质量发展的道路上披荆斩棘、阔步向前。必须始终坚持创新为要。创新是引领发展的第一动力。要突破思维定势，摆脱路径依赖，坚定下好创新“先手棋”，以创新培育新动能、抢占制高点、构建新优势。必须始终坚持生态为先。青山就是美丽，蓝天也是幸福。“两山”理论是我们必须遵循的根本方略，要下定“江拐弯、海调头”的决心，坚决打赢污染防治攻坚战，不断提高经济发展的“含金量”和“含绿量”。必须始终坚持民生为重。民生无小事，枝叶总关情。人民对美好生活的向往就是我们的奋斗目标，要直面难点痛点，多干群众叫好的实事好事，在共建共享中凝聚民心、赢得支持。必须始终坚持实干为本。真抓实干才能创造实绩。要把苦干实干当作人生坚守，把落细落实作为工作要求，明知征途有艰险，越是艰险越向前，努力干出一番无悔于新时代、无愧于铜陵人民的崭新业绩。

　　各位代表！幸福都是奋斗出来的。一年奋斗之路，就是一曲奋进之歌。令人感铭于心、难以忘怀的是：习近平新时代中国特色社会主义思想科学指引、掌舵领航，省委省政府深切关怀、深情关爱，指明了铜陵高质量发展的康庄大道；市委统揽全局、科学决策，市人大和市政协全力支持、有效监督，汇聚了铜陵加快发展的磅礴力量；全市广大干部群众同心协力、奋发进取，广大在外乡贤回报家乡、鼎力相助，谱写着铜陵跨越发展的恢弘乐章。在此，我代表市人民政府，向全市人民，向各民主党派、各人民团体和各界人士，向驻铜解放军指战员、武警官兵和政法公安干警，向关心支持铜陵改革发展的省直各部门、兄弟城市和海内外友好人士，表示最衷心的感谢，致以最崇高的敬意！

　　各位代表！前进路上无坦途。我们清醒地认识到，作为典型的资源型城市，铜陵正处在转型升级、爬坡过坎的关键时期，还有不少“攻坚战”、“持久战”要打。全市经济发展质量还不够高，牵动性的大项目、好项目不多，科技创新和人才支撑不够有力，产业结构不优、新旧动能接续不足的矛盾较为突出。实体经济发展面临诸多挑战，部分企业生产经营困难，营商环境需要进一步优化。金融等领域风险隐患仍然存在，脱贫攻坚、污染防治任务艰巨。民生领域还有不少短板，化解社会矛盾需要继续努力。政府系统部分工作人员综合素养不能完全适应新时代新要求，改革意识、创新意识、服务意识、担当意识亟需增强，谋划力、执行力有待提升。面对这些矛盾和问题，我们愈加感受到，为者常成，行者常至，越是百端待举、千头万绪，越要坚定方向，满怀信心，锲而不舍抓落实，久久为功求实效。

**二、奋进2019，加速赶超、追求卓越**

　　2019年是新中国成立70周年，是全面建成小康社会的关键之年，做好今年政府工作意义重大。看宏观大势，世界正面临百年未有之大变局，我国发展仍处于并长期处于重要战略机遇期，长三角一体化发展上升为国家战略，利于我们应势而谋；观区域态势，全省经济稳中向好，省委、省政府明确支持资源型城市转型升级，需要我们顺势而动；察自身趋势，基础设施持续改善，产业项目加速集聚，简政放权深入推进，催促我们乘势而为。多重机遇叠加的铜都大地，必将呈现更足的发展韧性，迸发更强的创造活力。我们一定要坚定信心，抢抓机遇，积极作为，以新理念新坐标新视野推动各项工作，奋力开创新时代铜陵高质量发展新局面。

　　今年政府工作的总体要求是：高举习近平新时代中国特色社会主义思想伟大旗帜，深入贯彻落实党的十九大、十九届二中、三中全会精神，统筹推进“五位一体”总体布局，协调推进“四个全面”战略布局，全面落实省委、省政府和市委十届九次全会决策部署，牢牢把握并全面用好重要战略机遇期，坚持稳中求进工作总基调，坚持新发展理念，坚持推动高质量发展，坚持以供给侧结构性改革为主线，坚持深化市场化改革、扩大高水平开放，继续打好“三大攻坚战”，着力激发微观主体活力，扎实做好“六稳”工作，开拓创新，拼搏进取，与改革窗口期赛跑，与优秀城市的产业生长速度赛跑，与卓越之城的动能转换需求赛跑，培强转型发展新动能，提升城市核心竞争力，保持经济持续健康发展和社会大局稳定，加快建设现代化幸福美丽新铜陵，为全面建成小康社会收官打下决定性基础，以优异成绩庆祝新中国成立70周年。

　　今年经济社会发展的主要预期目标是：全市生产总值增长7%－7.5%；财政收入与经济增长同步；固定资产投资增长8%以上；社会消费品零售总额增长9%以上；城镇、农村居民人均可支配收入分别增长8%以上、9%以上；居民消费价格指数涨幅3.5%以下；城镇调查失业率控制在5%以内；完成省政府下达的节能减排年度目标任务。重点做好八个方面工作：

　　（一）开启建设“卓越之城”新篇章。坚持全球视野，对标国内一流，不断增强城市吸引力、创造力、竞争力，加快向卓越之城迈进。

　　建设智造铜陵。牢牢扭住“三重一创”重点，提升制造业产业链水平，推动铜陵制造向铜陵智造转变。发展壮大铜基新材料、新能源汽车及零部件、精细化工等新兴产业，争取半导体装备与材料入列省级重大工程或专项，战略性新兴产业产值占规上工业比重提升0.5个百分点。大力发展集成电路、5G产业、生物医药及仿制药、新能源、3D增材与制造、以金属铼为代表的高端新材料等现代先进制造，打造地标性产业。加速发展智能制造，推动百家规上企业登“云”，新增省级智能工厂、数字化车间6家，国家“两化”融合贯标企业15家，争创省级智能制造示范区。

　　建设数字铜陵。制定出台支持数字产业发展政策，推进数字经济与各领域之间的跨界融合。加快汇聚数据资源，推进“数字铜陵”中心建设，打通数据壁垒，强化数据集成，促进数据资源共用共享。加快打造数据平台，深化与科大讯飞、阿里云、腾讯的战略合作，建成“城市超脑”一期，扩大“工业大脑”试点示范，组建工业大数据公司，促进铜官数谷、大数据产业园集聚发展。加快推进数据应用，启动新一代互联网（IPV6）、5G网络建设，在全省率先完成政府网站IPV6升级，推进物联网广泛应用，在教育、医疗等领域再推出一批“数字+”“智慧+”应用项目，让数字经济更好地融入生活。

　　建设线上铜陵。在紧抓产业互联网的同时，补上消费互联网的课，树牢“买全球、卖全球”的理念，以咬定青山不放松的韧劲，推动电商产业爆发式发展，培育限上电商企业40家以上，全口径网上零售额增长40%以上。引进大型电商贸易集团、跨境电商平台来铜设立区域性或功能性总部，大力支持艺福江南、中盛画材、铜草花等电商企业做大做强，鼓励洁雅生物科技等制造业企业分离发展电商。抢抓机遇发展跨境电商，探索开展直购进口业务，支持企业建设境外跨境电子商务海外仓，建成跨境电商产业园。实施“互联网+”农产品出村工程，强化电商企业与小农户、家庭农场、农民合作社等产销对接，农产品网络销售额突破3亿元，打造一批示范镇村和网店。

　　建设知识铜陵。以争创国家知识产权示范市为抓手，大力发展知识经济，打造城市IP，新增国家级知识产权示范优势企业3家以上，每万人发明专利拥有量11件以上。扩大知识产权质押融资试点，加强知识产权管理体系建设，完成20户企业国家知识产权管理规范贯标，构建知识产权“护城河”。深入实施品牌战略和标准发展战略，新增国家、行业和地方标准5项以上，培育中国驰名商标、国家地理标志保护产品2个以上，形成更多的区域经济品牌。

　　（二）构筑区域“四大中心”新高地。传承城市优良基因，发挥产业、区位等独特竞争优势，提升皖中南中心城市辐射力。

　　打造皖中南科技创新中心。聚焦关键核心技术“卡脖子”问题，全面提升创新能力和效率，争创国家创新型试点市，全社会研发投入强度2.5%以上。开展创新主体培育工程，新认定高新技术企业、科技型中小企业120家以上，高新技术产业增加值增长12%以上。强化创新平台建设，落地运行中科大、北京矿冶等科创和技术转移中心，规上工业企业研发机构覆盖率达33%。聚焦集成电路、基础软件等产业，组织开展重大专项联合攻关10个。加强全生命周期创新链建设，争创一批国家和省级科技孵化器、众创空间。

　　打造皖中南金融服务业中心。强化金融风险防范能力建设，组建地方金融监管局，落实监管职责和风险处置责任，坚决守住不发生系统性金融风险底线。持续开展地方金融机构不良资产压降行动，重拳整治非法金融，严防发生企业债券违约事件。健全以政府债券为主体的举债融资体制，抢抓中央增加地方政府专项债券规模机遇，争取更多项目收益平衡的专项债，杜绝新增隐性债务。加强市属国有企业债务管理，加快市场化、法治化债转股。以城市金融化为方向，大力发展供应链金融等新兴业态，全力做好科讯金服、易酒批、瓜子养车等金融“新势力”落地服务。规范发展互联网金融，支持企业申报互联网金融牌照，引导金融机构与互联网企业合作创新金融产品。招引长三角一体化基金、兴业基金、洪泰资本等落户铜陵，做大基金规模和效益，打造铜陵“基金部落”，充分发挥基金和投资人对优质项目“牧羊人”作用。积极发展多层次资本市场，扩大直接融资范围和比例，鼓励企业通过发行企业债、公司债、中期票据、信托等进行融资，支持上市公司股权再融资，科学做好市值维护。全年新增IPO报会在审企业1户，辅导备案1户，省股权交易中心挂牌30户，培育一批科创板后备企业。

　　打造皖中南商贸物流中心。深化铜官区国家服务业综合改革试点，加快科技服务、工业设计、现代物流等生产性服务业发展，创建省级现代服务业集聚区1个以上，新增限上企业30家以上。适应居民消费升级新趋势，以新流通引领新零售，延展B2B、B2C/O2O、网络定制等新模式。发展汽车金融消费，培育名品名店名街。优化商贸物流设施空间布局，完善城乡快递服务网络，鼓励多式联动，发展冷链物流。完成枞阳桥港物流园、义安高铁物流园项目前期，开工建设国际有色金属物流园，布局建设砂石集散中心，新增规上物流企业5家、国家3A级以上物流企业3家。

　　打造皖中南大健康产业中心。完善健康医疗服务体系，推动市人民医院、市立医院扩大规模、强身健体，全市基层医疗机构全部实现标准化。引入“好医生”、迪安诊断、上海桐树等高端优质医疗资源，组建现代医疗集团。加快润泽医养中心等项目建设，建成运营石城医养康复中心，争创全省医养结合试点示范市。建成省级中医药健康旅游基地，构建省内一流大健康产业孵化中心。

　　（三）培育产业转型发展新动能。贯彻“巩固、增强、提升、畅通”八字方针，推动传统产业涅槃重生，大力培育新动能新产业，开辟产业转型新境界。

　　赋能传统产业升级。开展新一轮技改升级工程，推动传统产业向“微笑曲线”两端延伸，向价值链高端跃升。实施亿元以上技改项目50个以上，技改投资占工业投资比重60%以上。支持龙头骨干企业聚焦主业开展并购重组，助推铜陵有色跻身世界500强。深入实施“优质工程”企业诊断计划，深化与工信部赛迪研究院、电子五所合作，帮助企业破瓶颈、增效益。全面推进质量提升行动，新增省级工业精品、新产品25个以上，争创全国质量强市示范市、制造业标准化示范市。

　　催生新兴增长点。坚持“无中生有”“有中生新”，实施新经济培育计划，引进地标性企业，点燃发展新引擎。构建发展新生态。优化产业生态圈，搭建优秀的承载平台，让投资主体充分选择、自由落户。集聚优质人力资源，厚植要素价格优势，打造“成本洼地”。引进企业新物种。抢占时代风口，积极发展众创、众包、众筹等新模式，加快发展共享、智慧、平台经济等新业态。办好国际铜加工协会技术年会、长三角国家公共文化服务体系示范区合作机制大会，积极争取承办各类行业和专业高端展会、论坛，力争月月有活动、季季有节会，努力实现办好一个会、活跃一座城，带来一批项目。锻造精准新服务。不断优化公共服务，对所有涉及市场准入的行政审批事项按“证照分离”模式进行分类管理，持续推进政务服务“一网、一门、一次”改革，探索推行告知承诺制，让企业和群众到政府办事像“网购”一样方便。精准实施个性服务，聚焦企业差异化诉求，提供点单式定制服务，当好“五星级服务员”。

　　提高项目建设质效。坚持把项目作为发展的生命线、主抓手，深入落实高质量“四督四保”制度，全年安排亿元以上项目400个，开工90个、竣工80个，完成投资330亿元以上。突出抓好产业项目建设，推动泰富碳新材料、中建材环保装备制造、连浩通精密大电流接触件等项目开工建设，加快北大青鸟氮化镓晶圆片、有色二次资源回收利用等项目建设进度，确保信敏惠新能源汽车电池溶剂、万华秸秆综合利用等项目竣工投产。力推重大基础设施项目建设，开工建设G3长江公铁大桥，配合推进引江济淮工程，完成国道236改建，做实铜枞宜市域铁路、铜九黄城际铁路、开发区过江通道和胥坝夹江大桥等项目前期。围绕“公铁水”、生态环保等基础领域，以及人工智能、工业互联网等新型基础设施，谋划储备一批重大项目，争取更多项目纳入国家和省盘子。

　　（四）激发全面改革开放新活力。用足用好改革开放40周年系列政策红利，改革不停顿，开放不止步，努力在新时代改革开放再出发中展现铜陵风采。

　　推动重点改革走深走实。完成市、县（区）政府机构改革，深入推进事业单位分类改革。深化国资国企改革，积极推进混合所有制改革，支持铜陵有色产权多元化改革、加快集团整体上市步伐，完成铜化集团增资扩股，推动投融资法人主体市场化转型；优化国有资本布局，开展国有资本投资公司、运营公司试点建设；单设市国资委，健全以管资本为主的国有资产管理体制。完善投融资体制，鼓励社会资本参与基础设施、公共服务等重点领域建设，大力推进PPP项目。持续深化社区改革，探索建立“社区吹哨、部门报到”机制，开启基层治理新模式。推进教育体制改革，依法下放初中阶段教育管理权至区级。深化医药卫生体制改革，巩固紧密型医联体改革试点成果，全面推行县域医共体改革。

　　推动民营经济做大做强。坚持把民营企业和民营企业家当作“自己人”，组建民营经济促进局，推动各项政策落地见效，为民营经济发展清障护航。深入实施民营经济提升工程，新进规上企业45家以上，民营经济增加值占比提升到51.8%。推进中小企业梯度培育工程，争创“专精特新”企业40家以上，培育一批“单打冠军”和“排头兵”企业。严格落实减税降费政策，实质性降低企业制度性交易成本、要素成本。设立8亿元以上民营企业纾困救助基金，鼓励金融机构落实续贷政策、扩大信贷投放，着力破解融资难融资贵问题。常态化推进“四送一服”双千工程，深入开展民营企业大走访，健全企业家参与涉企政策制定机制，依法保护企业家人身和财产安全，构建亲清新型政商关系，让企业在铜都大地创业愉快、兴业愉快、发展愉快。

　　推动城市更加开明开放。坚持小城市、大格局，敞开怀抱，开放包容，着力打造自由无障碍城市。全面对接长江经济带、粤港澳大湾区、京津冀等国家重大战略，深度参与长三角一体化发展，链接上海、苏州、杭州、深圳等中心城市，加强互联互通和区域协作，为未来生长奠基布局。复制推广自贸区改革试点经验，争取纳入安徽自由贸易试验区申报范围，建成运营皖中南B型保税物流中心，推进铜期货交割仓库申建，推动“圈区管理”园区转型发展。深入实施“双招双引”首位工程，推进精准招商、产业链招商、以商引商，跟紧盯牢总投资千亿元的新能源关键材料产业园、总投资百亿元的北大青鸟半导体产业园等一批重点项目，新引进亿元以上项目165个，其中10亿元以上项目12个，实际到位内资580亿元，实际利用外资3.2亿美元。大力实施“铜都英才计划”，用好股权激励、柔性引进等政策，构建人才“强磁场”，招引高层次科技人才团队10个以上。深度融入“一带一路”倡议，新增进出口实绩企业35家以上，保持外贸稳定向好态势。

　　推动开发园区升级升温。出台支持开发区高质量发展实施意见，优化开发园区管理体制，全面推行工业项目“承诺即开工”，推动开发园区差异化、特色化发展，打造全市经济增长的最高地。铜陵经开区要发挥担纲承梁作用，完成工业总产值1180亿元以上，构建全省具有较强竞争力的经济板块；枞阳开发区要聚力加强横埠工业园建设，促进基础设施提档升级，壮大万华板块、汽车零部件配套等重点产业；狮子山高新区要聚焦“高”“新”做文章，培育发展人工智能、大数据等产业；义安开发区要依托国家级循环化改造示范试点园区，大力发展新材料、装备制造、电子信息等主导产业；郊区开发区要创造条件扩区扩容，大力发展口岸经济，推进专业化电商园、临港物流园建设。市直各部门、各县区要把开发区当作人生奋斗的战场、重点服务的对象，集全市之力奏响开发区高质量发展最强音。

　　（五）打造生态文明建设新样板。坚持生态优先、绿色发展，大力呵护“山水林田湖草”生命共同体，让绿色成为发展主色调。

　　建设美丽长江（铜陵）经济带。围绕“四美四高”，强力实施“十大行动”，推动139项重点任务有效落实。深入推进沿江1公里化工企业综合整治，完成“散乱污”企业整治。加强入江排污口治理，全面完成规范化改造和在线监测设施建设，努力实现“清水入江”。启动滨江生态公园2.1公里南延伸段建设，扩大江北近岸区绿化覆盖面，打造滨水生态廊道。

　　打好污染防治攻坚战。聚焦“三大保卫战”，加大工作和投入力度，确保生态环境质量不断改善。扎实开展“五控”行动，推动重点行业企业超低排放改造，开展柴油货车污染专项整治，保持治超治限常态化，让良好“气质”多起来。强力整治横港地区突出环境问题，实施工业企业大气污染双减工程，推动烟囱“去白化”改造，加大美化彩化力度，打造“高颜值”生态画卷。严格落实河长制、湖长制，完成市三水厂迁建，改扩建城市污水处理设施，启动农村黑臭水体治理，促进城区黑臭水体“长制久清”。加强土壤污染管控与治理，实现重点重金属污染物排放“零增长”。以“不贰过”的态度狠抓中央、省环保督察反馈问题整改，确保改彻底、改到位。探索环保管家模式，保持环境监管执法高压态势。

　　厚植城市绿化景观带。划定永久性城市绿带，推进全域增绿提质，扩大绿色生态空间。深入实施林长制，开展大规模国土绿化行动，完成营造林8.3万亩。持续开展“增绿补绿、增花添彩”绿化提升行动，建设“东方薰衣草”—铜草花主题公园，建成口袋公园4个，新增和改造提升绿化140万平方米，打造“繁花似锦、四季多彩”的花园铜都。全面落实矿山山长制，推进绿色矿山建设，恢复矿山裸露山体植被400亩，不断彰显城市绿色之美。加强西湖湿地及周边水环境治理，擦亮新区生态名片。

　　争创无废城市试点市。以“趋零排放”为愿景，推进固体废物源头减量和资源化利用，加快建设无废社会。推动生活垃圾分类试点扩面，建设城市生活垃圾转运和环卫一体化项目，建成固废综合开发再利用、建筑垃圾转运处置项目。深化省级以上园区循环化改造，建设开发区集中供热等循环经济项目，与中节能合作共建国家循环利用基地，打造循环经济升级版。

　　（六）构建城市建设管理新格局。实施新一轮城市总体规划，扩规模、提能级、增品位，彰显现代化滨江城市精致大气之美。

　　以一流标准建设新区。坚定不移推动教育、医疗等优质资源延伸覆盖，推进产城融合、人气集聚，加速西湖新区蝶变腾飞。启动市人民医院西湖分院建设，建成十五中西湖校区、师范附小新校区主体工程，打造示范全国的社区邻里中心，让居民就近享受最好的配套服务。创新思路启动江北新区建设，打破区划界限规划核心区，超前谋划氢能源等支柱产业，开工建设江北港一期和江北港铁路专用线，打造未来发展的重要增长极。强化东部、南部城区配套建设，提升综合承载功能。

　　以工匠精神更新城市。深入实施城市“双修”，建设一批精品便民工程，彰显城市魅力。舒展城区路网经脉，开工建设义安大道北延伸段高架桥及二冶专用线改造工程，打通翠湖二路东延伸段、铜官大道北延伸段、乌木山大道等一批断头路、丁字路，建成城市微循环道路10公里以上。优先发展公共交通，再投放一批清洁能源公交车，推进公交首末站及站亭改建工程，力争成为省公交示范市。实施城市微改造，兴建人民一路、长江二路等一批停车设施；开工地下综合管廊3条，新改建燃气管网35公里、供水管网30公里；完成老旧小区整治9个，加装多层住宅电梯20个单元以上，试点建设集中供暖小区，让市民居住更舒适、生活更舒心。

　　以绣花功夫管理城市。坚持从点滴之处营造美感，让城市经得起品味、耐得住细看。规范出租车、网约车经营行为，建设“信用交通市”。深入开展全城“大清洗”活动，加强犬类规范管理，大力整治占道经营、非法广告、违章停车，打造路畅、街净、人和、景美的宜居城市。推行老旧小区准物业管理，推动集中行政执法进小区，让城市工作更高效、更暖心。

　　（七）推动乡村振兴取得新突破。坚持农业农村优先发展，大力实施乡村振兴战略规划，推动农业全面升级、农村全面进步、农民全面发展。

　　壮大县域经济实力。坚定跨江联动发展，加大对枞阳重大基础设施建设支持力度，开工建设合枞高速公路、国道237会宫至官桥段，实施省道320会宫至横埠段改造，建成省道335白湖至麒麟段。继续实施枞阳县城防洪保安等工程，补齐水利设施短板。支持枞阳争创省级县域特色产业集群基地，加快打造新材料、智能家居制造“双百亿”产业集群。提升县城建管水平，推动横埠等乡镇建设经济发达镇，打造一批工业强镇、农业重镇、旅游名镇，增强对农业转移人口的吸附力。

　　放大现代农业效应。落实藏粮于地、藏粮于技战略，坚守“农地姓农”底线，建设高标准农田5.8万亩。全面开展农产品加工业提升行动，推动水稻、牡丹、食用菌全产业链开发，谋划设立市级农业产业园3个，规上企业农产品加工业产值增长8%以上，新增农业产业化联合体8个、家庭农场150个。支持枞阳县建设国家田园综合体，义安区创建国家农业高新技术产业示范区，普济圩农场争创国家级稻渔综合种养示范区。

　　改善农村人居环境。推进镇村建设，促进大通、钟鸣等特色小城镇形态塑造、业态提升、生态改观，建成美丽乡村省级中心村22个、市级中心村14个、特色自然村9个。推动农村基础设施一体规划、互联互通，建成“四好农村路”800公里，完善农村电网设施，开工建设排涝泵站10个，改造农村危房1400户，再解决2万农村居民饮水安全问题。学好用活“浙江经验”，启动“五清一改”清洁家园行动，持续推进农村环境“三大革命”，完成1.85万户常住农户卫生厕所改造，建成9个乡镇政府驻地生活污水处理设施，生活垃圾无害化处理率达80%，实现“门口山水净，村头远客来”。

　　深化农业农村改革。持续深化农村“三权分置”改革，扎实推进土地经营权贷款抵押试点，激活“沉睡”的土地资源。认真开展“清产核资”回头看，完成100个村级集体经营性资产股份合作制改革、50个村“三变”改革试点，促进农村集体经济发展和农民增收。精心培育一批农业职业经理人、乡村工匠，鼓励和吸引各类人才投身农村广阔天地。

　　（八）竭力满足人民群众新期盼。坚持以群众需求为导向，高质量实施省市民生工程，努力让市民的生活一年更比一年好。

　　强力推进脱贫攻坚。立下愚公之志，倾注洪荒之力，打好年度精准脱贫攻坚战，确保1.1万以上贫困人口实现脱贫，剩余10个贫困村全部出列。实施“四送四通”扶贫直通车，大力推进“四带一自”产业扶贫等特色扶贫模式，完善“351”“180”健康脱贫政策，全面落实教育扶贫政策，打牢“两不愁三保障”底层，不断提高脱贫质量。突出扶贫政策衔接，制定收入水平略高于建档立卡贫困户的群体专门扶持政策，实现非贫困村贫困人口帮扶措施全覆盖。

　　坚守民生保障底线。把稳就业摆在更加突出位置，加大高校毕业生、退役军人等重点群体就业创业扶持力度，新增就业2.5万人以上。深入实施全民参保计划，扩大新业态就业人员社会保险覆盖面，实现枞阳县城乡居民基本医疗保险并轨运行。发展社会救助、慈善和残疾人事业，提高城乡低保和特困供养人员生活保障标准。加快养老服务三级中心建设。持续开展棚改攻坚行动，改造棚户区8000户，全面完成宝山区域整治。坚持“房子是用来住的、不是用来炒的”定位，促进房地产市场平稳健康发展。

　　织密公共服务网络。实施第三期学前教育行动计划，新改建公办幼儿园17所，鼓励引导规范社会力量办园。推动义务教育优质均衡发展，启动北斗星城九年一贯制学校、四中新校区建设。深化“名校+”战略，筹建北师大铜陵学校。实施现代职业教育质量提升工程，组建大江职业教育集团，打造全国知名职教高地。支持铜陵学院建设地方应用型高水平大学。办好群众性文化活动。持续推进天井湖国家级旅游度假区建设，支持大通古镇、浮山争创5A级景区，力争枞阳县创成国家全域旅游示范县。培育践行社会主义核心价值观，广泛开展群众性精神文明创建活动。提高市民科学素质。建设体育强市，扩大全国全民健身示范市品牌效应。

　　构筑社会善治格局。提升城乡社区治理能力，推进义安区全国首批农村社区治理实验区建设。常态化开展“铸安”行动，有效防范较大以上安全生产事故。强化食品药品安全监管，提升重大动物疫病防控水平。加强应急管理，提高综合防灾减灾救灾能力。完善预防和化解社会矛盾机制，引导信访问题在法治轨道妥善解决。深化“平安铜陵”建设，纵深推进扫黑除恶专项斗争，巩固全国禁毒示范城市创建成果。扎实做好第四次全国经济普查。支持工会、共青团、妇联等人民团体广泛参与社会治理，做好民族宗教、新闻出版、广播电视、融媒体、地方志、地震、气象、档案等工作。

**三、奋勇担当，建设人民满意的服务型政府**

　　新时代是奋斗者的时代。我们一定要扛起新担当、展现新作为，以政治建设为统领，以机构改革为动力，全面加强政府自身建设，同心书写让人民满意的“新时代答卷”。

　　（一）打造担当实干的“施工队”。树牢“四个意识”，坚定“四个自信”，坚决做到“两个维护”，扎实开展“不忘初心、牢记使命”主题教育，始终在思想上政治上行动上同以习近平同志为核心的党中央保持高度一致，不折不扣贯彻落实党中央国务院、省委省政府和市委决策部署。注重向企业家、银行家、科学家学，学习人工智能、数字经济、现代金融等新知识，全面提升施工队员的专业知识和能力素养，牢牢把握工作主动权。强化“以能力比高低、以实绩论英雄”的鲜明导向，突出效率优先，及时准确地将市委部署转化为举措，将蓝图变为行动，将梦想化为现实。增强斗争精神，拿出舍我其谁的气魄，努力奔跑、加速冲刺，在实干中创造新业绩，在担当中彰显新形象，在奋斗中谱写新辉煌。

　　（二）争当依法行政的“践行者”。全面落实常态化学法制度，加强重大事项合法性审查，发挥政府法律顾问作用，提高政府立法质量，提升政府决策科学化、民主化、法治化水平。深化综合行政执法改革，加快推进机构、职能、权限、程序、责任法定化，进一步规范公正文明执法。全面推进政务公开，自觉接受人大法律监督、政协民主监督、舆论监督和社会各方面监督，认真听取各民主党派、工商联、无党派人士和人民团体意见，让人民监督权力，让权力在阳光下运行。

　　（三）勇做服务发展的“店小二”。牢固树立“服务就是义务、服务就是效益”理念，用心当好服务企业、服务群众的“店小二”， 推行“保姆式”服务，让最优服务成为铜陵发展永不褪色的金字招牌。弘扬“马上办”的作风，以“今天再晚也是早、明天再早也是晚”的精神，绷紧思想、抓紧时间、拧紧工作，持续治理“庸懒散”，坚决打通“中梗阻”，齐按发展“快车键”，将任务抓实抓牢，让发展提速提效。学习借鉴上海经验，聘任市政务首席“找茬官”，聚焦经济社会发展的痛点、阻点，围绕群众关切的热点、难点，找问题、提建议、谋对策，做到“有求必应，无事不扰”。

　　（四）当好遵规守纪的“排头兵”。扛起扛牢全面从严治党主体责任，全面落实意识形态责任制。深入贯彻中央八项规定精神及省、市委实施细则，坚持不懈纠“四风”、正作风，严格规范督查检查考核，让基层把更多时间用在谋发展、抓落实上。激励新时代新担当新作为，旗帜鲜明为担当干事者鼓劲撑腰，对懒政怠政者严肃问责。认真履行“一岗双责”，加强工程建设、土地出让、政府采购等重点领域监督管理，推进审计监督全覆盖，严肃查处侵害群众利益的突出问题，巩固发展反腐败斗争压倒性态势，让清正廉洁成为干部自觉追求，让干事创业在铜都大地蔚然成风。

　　各位代表！

　　乘风破浪潮头立，奔跑追梦正当时。让我们更加紧密地团结在以习近平同志为核心的党中央周围，在省委、省政府和中共铜陵市委的坚强领导下，只争朝夕，砥砺前行，挥洒汗水，拼尽全力，奋力开创现代化幸福美丽新铜陵建设新局面，以优异成绩庆祝新中国成立70周年！
